# Supplementary material for: The causal role of circulating inflammatory markers in osteoporosis: a bidirectional Mendelian randomized study
Source: Front Immunol. 2024 Jul 18;15:1412298. doi: 10.3389/fimmu.2024.1412298 (PMC11291241; doi:10.3389/fimmu.2024.1412298)
Supplement: Supplementary file 1 [file DataSheet_1.zip › supplementary material/OSTEOPOROSIS-CIM/GCST90274813.tsv.gz_leave_one_out.pdf]

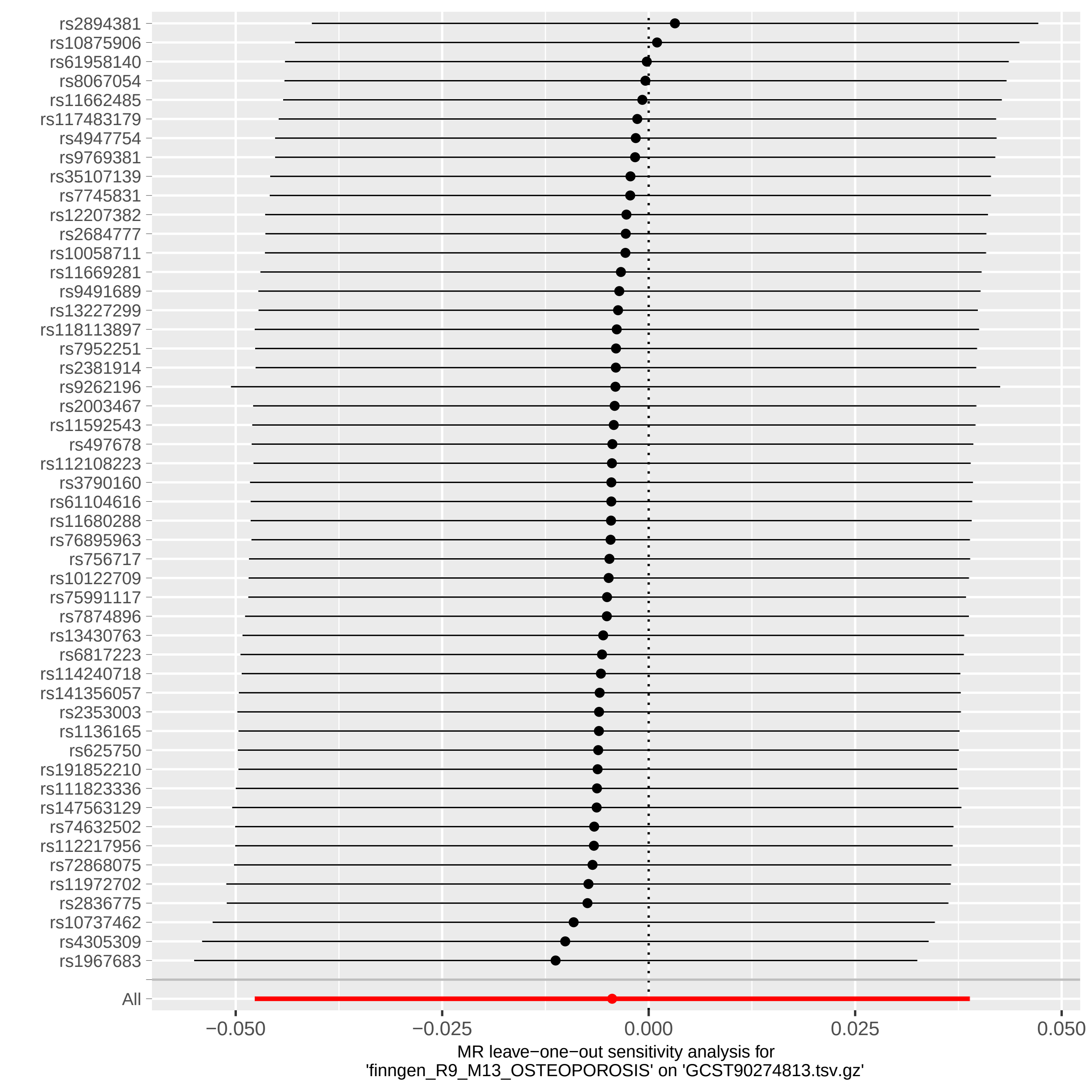
rs2894381
rs10875906
rs61958140
rs8067054
rs11662485
rs117483179
rs4947754
rs9769381
rs35107139
rs7745831
rs12207382
rs2684777
rs10058711
rs11669281
rs9491689
rs13227299
rs118113897
rs7952251
rs2381914
rs9262196
rs2003467
rs11592543
rs497678
rs112108223
rs3790160
rs61104616
rs11680288
rs76895963
rs756717
rs10122709
rs75991117
rs7874896
rs13430763
rs6817223
rs114240718
rs141356057
rs2353003
rs1136165
rs625750
rs191852210
rs111823336
rs147563129
rs74632502
rs112217956
rs72868075
rs11972702
rs2836775
rs10737462
rs4305309
rs1967683
All
−0.050
−0.025
0.000
0.025
0.050
MR leave−one−out sensitivity analysis for
'finngen_R9_M13_OSTEOPOROSIS' on 'GCST90274813.tsv.gz'
